# Looking into nerve damage in the cornea

**DOI:** 10.7554/eLife.51497

**Published:** 2019-10-08

**Authors:** Mihaela Gadjeva

**Affiliations:** 1 Division of Infectious Disease, Department of MedicineBrigham and Women’s HospitalBostonUnited States; 2 Harvard Medical SchoolBostonUnited States

**Keywords:** cornea, neuropathy, inflammation, HSV-1, graft vs. host disease, complement, Mouse

## Abstract

Interactions between T helper cells and the complement system promote loss of sensory neurons in the eye.

**Related research article** Royer DJ, Echegaray-Mendez J, Lin L, Gmyrek GB, Mathew R, Saban DR, Perez VL, Carr D. 2019. Complement and CD4^+^ T cells drive context-specific corneal sensory neuropathy. *eLife* **8**:e48378. doi: 10.7554/eLife.48378

The cornea is likely the most densely innervated part of the human body with approximately 7,000 sensory neurons per square millimeter, making it 300 to 600 times more sensitive to stimuli than the skin (Siran et al., 2018; Cruzat et al., 2017; González-González et al., 2017). The majority of neurons in the cornea are nociceptors: they respond to noxious stimuli, such as touch, temperature, and toxins, by signaling pain.

Infections and other inflammatory conditions, such as graft versus host disease (where the host immune system attacks transplanted tissue), are often associated with neuropathies. For example, mice with a herpes simplex virus type 1 (HSV-1) infection in the eye stop perceiving mechanical stimuli due to nerve loss. However, the molecular mechanisms that govern neuropathy are still unknown. Understanding which molecules initiate the process could lead to new therapies for targeted pain management.

Infectious and non-infectious responses to inflammation often activate the complement system – an evolutionarily conserved system with over 30 soluble and membrane-associated proteins that enhance the ability of the immune system to clear infection. The complement system is usually activated by antibodies bound to the surfaces of pathogens such as viruses, bacteria or fungi, and it self-assembles through a well-coordinated cascade of proteolytic events. Every activation of the complement system eventually leads to the proteolytic cleavage of a protein called C3. Once cleaved, C3 undergoes a conformational change, exposing an active site that allows it to covalently bind to target surfaces. Labeling of a pathogen by C3 is a unique event that tags the target for clearance (Gadjeva et al., 1998).

The activation of the complement system occurs within seconds of a pathogen being recognized, and while its main purpose is to control pathogen spread, additional activities have been discovered. Now, in eLife, Derek Royer and colleagues at Duke University Medical School and the University of Oklahoma Health Sciences Center report on the effects of complement activation on the nerves in the cornea (Royer et al., 2019).

Royer et al. first showed that, in contrast with wild type mice, mice deficient for C3 did not lose mechanosensation in the cornea when infected with HSV-1. Consistent with this, these mice did not suffer nerve loss. These data illustrate that C3-dependent inflammatory processes lead to neuronal loss.

Next, Royer et al. showed that mice lacking T cells (a type of immune cell) did not lose sensation in the cornea when they were infected with HSV-1, despite having wild-type C3 protein. T helper cells are a type of T cell that produces proteins called cytokines to recruit other immune cells to the site of infection. When T helper cells were transferred from wild-type or C3 mutant mice infected with HSV-1 into mice without T cells, these mice progressively lost nerve endings in the cornea. This demonstrates that nerve and sensation loss in the cornea associated with HSV-1 infection require T helper cells.

Collectively, these data show that coordinated C3-dependent and T helper cell-dependent pathways are responsible for the loss of sensation in mice infected with HSV-1, although the exact mechanisms remain to be described.

Royer et al. also found that using drugs to prevent activation of the complement system reduced nerve damage upon HSV-1 infection, suggesting that the complement cascade could be a target in efforts to control neuronal integrity. Importantly, similar pathways are operational in a mouse model of graft versus host disease, where inhibiting the activation of complement rescued perception of mechanical cues and reduced neuronal loss. Finally, Royer et al. established that the C3 that caused tissue damage in the cornea was made by local myeloid and non-hemaotopoietic cells upon acute HSV-1 infection.

Together, these findings provide a strong foundation for future experiments to determine the possible molecular mechanisms linking the activation of the complement system and neuropathy (Figure 1). One hypothesis is that T helper cells in the cornea produce a cytokine called interferon: this causes myeloid cells to produce C3, resulting in neuron damage downstream. Another possibility is that products of complement cleavage stimulate macrophages to release neuronal growth factors, inducing overactivation of the Transient Receptor Potential Cation Channel Subfamily V Member 1 (TRPV1) in nociceptors, which leads to nerve damage (Shutov et al., 2016; Danigo et al., 2014). Alternatively, the complement system may induce assembly of the membrane attack complex on host cells. This complex usually attacks bacteria, but it might also damage the membranes of neurons.

**Figure 1.**
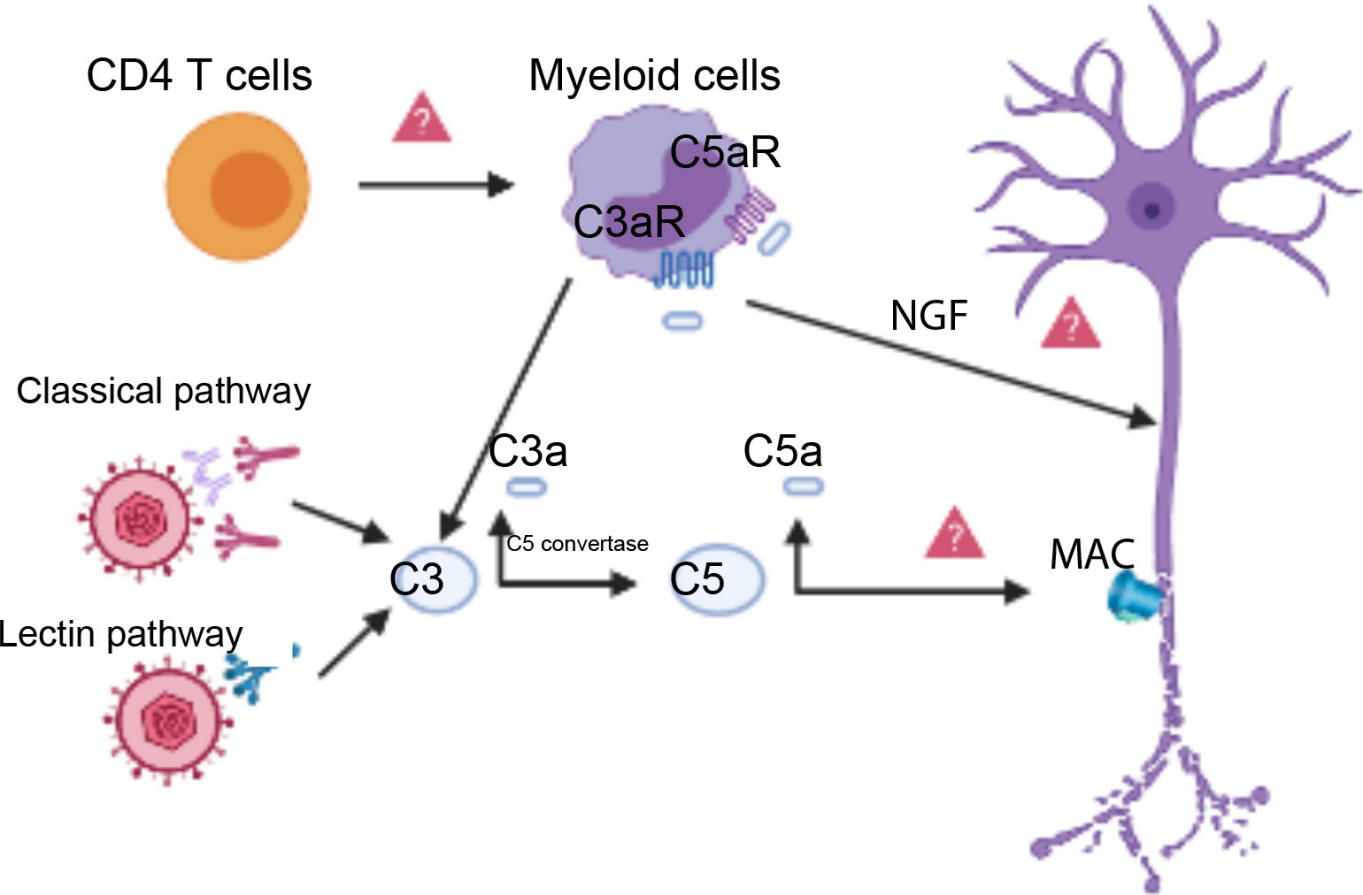
Complement activation triggers neuronal damage. The classical pathway or the lectin pathway can both activate the complement system (left) resulting in a proteolytic split of C3, which releases C3a and C3b. C3b (not shown) becomes a part of an enzyme known as the C5 convertase, which cleaves another complement protein called C5, releasing C5a and C5b. C5b (not shown) participates in the assembly of the membrane attack complex (MAC), which can lead to nerve damage when it forms on neuronal membranes (right). Complement cleavage products C3a and C5a can bind to the C3a receptor (C3aR) and the C5a receptor (C5aR) on myeloid cells called macrophages (top center), potentially inducing the synthesis of neuronal growth factors (NGF). NGF production could cause nerve damage through the overactivation of TRPV1 on sensory neurons. T helper cells (CD4 T cells, top left) coordinate with C3 to damage nerve cells upon HSV-1 infection. This coordination may happen through T helper cells producing interferon, which then induces myeloid cells (top center) to synthesize local complement proteins.

Today, there are more than 20 candidate drugs that block different aspects of complement activation. Understanding at which level the complement cascade has to be blocked to reduce the onset of nerve damage will ensure that existing therapies are applied more effectively, allowing faster translation to patients.
